# Understanding randomized controlled trial generalizability through an embedded molecular diagnostics trial

**DOI:** 10.1093/jncics/pkag040

**Published:** 2026-04-24

**Authors:** Patrick Lewicki, Arnav Srivastava, Ralph Jiang, Anna Johnson, Khurshid Ghani, Kevin Ginsburg, Tudor Borza, Kristian Stensland, Simpa S Salami, Elai Davicioni, Rodney L Dunn, Stephanie Daignault-Newton, Ganesh S Palapattu, Daniel E Spratt, Michael Cher, Matthew Schipper, Robert T Dess, Todd M Morgan, Udit Singhal

**Affiliations:** Department of Urology, University of Michigan, Ann Arbor, MI, United States; Department of Urology, University of Michigan, Ann Arbor, MI, United States; Department of Biostatistics, University of Michigan, Ann Arbor, MI, United States; Department of Urology, University of Michigan, Ann Arbor, MI, United States; Department of Urology, University of Michigan, Ann Arbor, MI, United States; Department of Urology, Wayne State University, Detroit, MI, United States; Department of Urology, University of Michigan, Ann Arbor, MI, United States; Department of Urology, University of Michigan, Ann Arbor, MI, United States; Department of Urology, University of Michigan, Ann Arbor, MI, United States; Veracyte, Inc, South San Francisco, CA, United States; Department of Urology, University of Michigan, Ann Arbor, MI, United States; Department of Urology, University of Michigan, Ann Arbor, MI, United States; Department of Urology, University of Michigan, Ann Arbor, MI, United States; Department of Radiation Oncology, University Hospitals Seidman Cancer Center, Cleveland, OH, United States; Department of Urology, Wayne State University, Detroit, MI, United States; Department of Biostatistics, University of Michigan, Ann Arbor, MI, United States; Department of Radiation Oncology, University of Michigan, Ann Arbor, MI, United States; Department of Radiation Oncology, University of Michigan, Ann Arbor, MI, United States; Department of Urology, University of Michigan, Ann Arbor, MI, United States; Department of Urology, University of Michigan, Ann Arbor, MI, United States

## Abstract

**Background:**

Issues with randomized controlled trial generalizability are well described, but whether these issues result from differences in patient treatment across contexts remains unknown. We studied treatment of patients with high-risk prostate cancer after radical prostatectomy inside and outside a randomized controlled trial evaluating the impact of a genomic classifier on post–radical prostatectomy treatment decision making (Genomics in Michigan Impacting Observation of Radiation [G-MINOR]; ClinicalTrials.gov identifier NCT02783950).

**Methods:**

G-MINOR enrolled 338 patients; propensity score–matched eligible but unenrolled patient cohorts (pretrial and trial contemporary) from the Michigan Urological Surgery Improvement Collaborative (MUSIC), in which the trial was embedded, were compared for rates and time to secondary treatment (adjuvant or salvage therapy) after prostatectomy.

**Results:**

Among 338 patients in the G-MINOR cohort, 69 (31 adjuvant, 38 salvage) received secondary treatment compared with 266 (183 adjuvant, 83 salvage) and 104 (60 adjuvant, 44 salvage) in the 1014 contemporary and 338 pretrial–matched MUSIC cohorts. Time to secondary treatment was much shorter in the MUSIC cohort across all comparisons. For example, matching G-MINOR to synchronous MUSIC patients demonstrated 84% vs 74% estimated 2-year treatment-free survival for trial and real-world patients, respectively (*P* < .001).

**Conclusions:**

Controlling for key clinicopathologic factors, patients in the G-MINOR randomized controlled trial and MUSIC cohorts were treated differently, even after stratifying by genomic risk. These findings suggest that challenges in randomized controlled trial generalizability extend beyond the representativeness of trial participants. Differences in management may also explain why divergent patient outcomes are observed in randomized controlled trials vs real-world settings.

## Introduction

Although randomized controlled trials represent the cornerstone of evidence-based medicine,^1^ issues with generalizability to real-world settings have been described.^2^^,^^3^ These issues may be due to the representativeness of a study population^4-11^ or to unmeasured influences such as the Hawthorne Effect, which proposes that direct observation changes human behavior^12^ and suggests that health care in the context of a randomized controlled trial may not resemble that of routine clinical practice.^13^ Whether observed discrepancies are the result of differences in data capture (ie, fastidious in trials yet limited in retrospective approaches) or to true divergence from real-world practice is largely unknown, but opportunities for the assessment of a clinician’s behavioral differences across randomized and nonrandomized controlled trial settings are sparse. Data that minimize the aforementioned limitations (eg, through clinically and geographically equivalent populations) would facilitate the assessment of differences in clinician behavior that may threaten randomized controlled trial generalizability.

Here, we use data from the recently published Genomics in Michigan Impacting Observation or Radiation (G-MINOR; ClinicalTrials.gov identifier NCT02783950) trial^4^ and Michigan Urologic Surgery Improvement Collaborative (MUSIC) to explore the extent to which treatment of trial patients resembles real-world practice by examining secondary therapy decisions after radical prostatectomy, the trial’s primary endpoint. Importantly, G-MINOR is nested within the infrastructure of MUSIC, a statewide collaborative consisting of community, hybrid, and academic urology practices that maintains a prospective clinical registry across numerous urologic disease spaces, including localized prostate cancer.^14^ Thus, trial (G-MINOR) and nontrial patients have mutual clinicians and identical data-collection infrastructure. This embeddedness affords a unique ability to more accurately probe the association between clinical trial participation and care delivery outcomes by controlling for differences in data capture or clinician characteristics. In this study, we tested the null hypothesis that patients enrolled in G-MINOR were treated similarly (vis-à-vis post–radical prostatectomy secondary treatment) to patients eligible but not enrolled in the trial because of chronicity (ie, eligible before trial start) or for other reasons (eg, not offered or declined to participate). Attempting to control for differences between trial and real-world populations brings us closer to an accurate estimation of the impact of trial participation on patient treatment and, potentially, generalizability.

## Methods

### Patient cohorts

**Figure 1.**
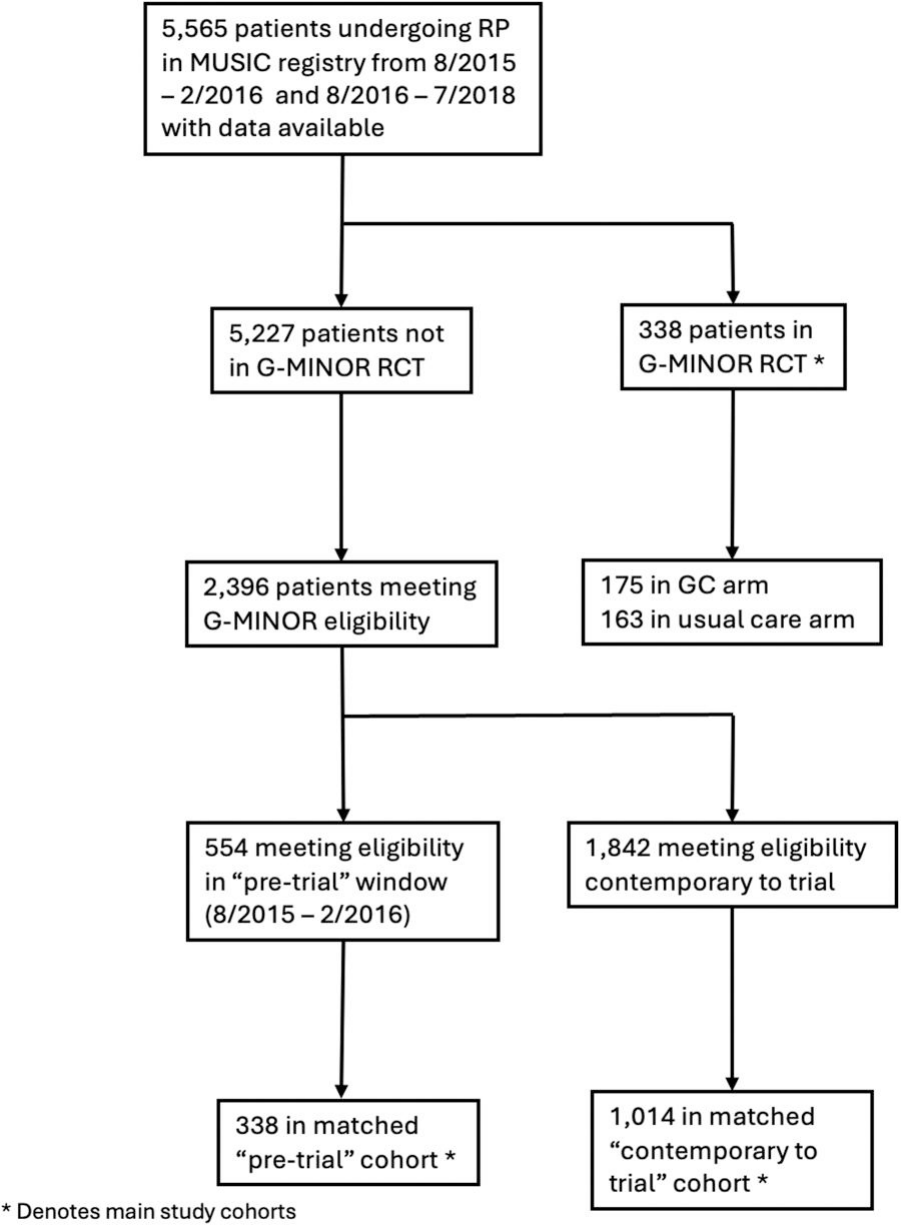
CONSORT diagram. G-MINOR = Genomics in Michigan Impacting Observations of Radiation; MUSIC = Michigan Urological Surgery Improvement Collaborative. ^a^Main study cohorts.

This study consisted of 2 main comparisons between patients enrolled in G-MINOR and, separately, 2 real-world cohorts of eligible but non-enrolled patients from MUSIC (Figure 1). In both comparisons, the real-world MUSIC cohort was drawn from clinical sites participating in G-MINOR, controlling for clinician-level factors. The first comparison included patients from MUSIC who were synchronous with or contemporary to G-MINOR and either were not offered the trial or declined to participate (hereafter, “matched MUSIC cohort”). Recognizing that this approach represents a potential source of unmeasured confounding because characteristics not captured in standard variables may influence the decision to participate in a trial, the second comparison draws its real-world cohort from patients who met eligibility in a window 9-13 months before initiation of trial enrollment (hereafter, “matched MUSIC pretrial cohort”). This time window was chosen given (1) 9 months of trial eligibility after radical prostatectomy and (2) potential underlying secular trends in the use of secondary treatment during this time, which necessitated temporal proximity to the trial’s start.

### G-MINOR

Tissue-based gene expression classifiers (eg, Decipher Prostate [Veracyte, Inc]) seek to improve stratification of patients at high risk of post–radical prostatectomy recurrence and assist with decision making regarding the need for post–radical prostatectomy secondary treatment with radiation therapy and androgen-deprivation therapy. G-MINOR was a prospective, multicenter, cluster-crossover randomized controlled trial evaluating the impact of post–radical prostatectomy genomic classifier testing (Decipher Prostate) plus a clinical risk score (Cancer of the Prostate Risk Assessment post-Surgical [CAPRA-S]) vs clinical risk score alone (“usual care”) on secondary treatment decision making.^15^ The trial enrolled patients at 12 MUSIC sites from January 2017 to August 2018. Trial eligibility criteria included radical prostatectomy within 9 months of enrollment, stage pT3 to 4N0 or positive surgical margins, and initial undetectable postoperative serum prostate-specific antigen (PSA) levels. Control arm patients underwent genomic classifier testing, but these results were not disclosed to patients or clinicians. The primary endpoint was receipt of adjuvant treatment (radiation therapy or androgen-deprivation therapy before biochemical recurrence [PSA ≥0.002ug/L] and within 18 months of radical prostatectomy).

### MUSIC

MUSIC is a statewide quality improvement collaborative consisting of more than 250 urologists across 46 diverse practices within the state of Michigan. MUSIC prospectively maintains a data registry across several urologic disease states, including prostate cancer. Patients eligible for the G-MINOR trial at 1 of the 12 G-MINOR sites but not enrolled in G-MINOR made up the 2 MUSIC (ie, real-world) cohorts. The first cohort consisted of patients contemporary to G-MINOR who were not offered or declined enrollment. The second cohort consisted of patients who met eligibility 9-13 months before trial initiation (August 2015–March 2016) for whom the trial was not available. To additionally control for varying patient characteristics, nearest-neighbor propensity score matching was performed on clinically important variables, including age; race; Charlson Comorbidity Index; pre–radical prostatectomy PSA level; and radical prostatectomy grade, stage, and surgical margin status.

### Trial interventions

The CAPRA-S score is a validated measure that estimates patient risk of biochemical recurrence following radical prostatectomy using routine clinical data (ie, PSA level, pathologic tumor stage, and pathologic tumor grade).^16^ CAPRA-S was provided to physicians for patients in both arms of the G-MINOR trial. Further, it is readily available as a free online resource to physicians treating patients outside the trial.

The Decipher Prostate test measures the expression of 22 genes to calculate a genomic risk score. The score estimates the 5-year and 10-year risk of metastasis as well as the 15-year risk of prostate cancer–specific mortality after radical prostatectomy.^17^ Within the trial, genomic classifier data were available only for patients in the genomic classifier arm, whereas within the matched MUSIC cohort, genomic classifier data were available whenever a physician ordered the test for a patient in routine, real-world clinical practice.

### Endpoints

Parallel to the G-MINOR trial, primary endpoints of the present study included receipt of secondary treatment (radiation therapy or androgen-deprivation therapy) and time from radical prostatectomy to secondary treatment (secondary treatment-free survival). Secondary endpoints included receipt of adjuvant and salvage treatment. Adjuvant treatment was defined as before biochemical recurrence and within 18 months of radical prostatectomy. Salvage treatment was defined as following biochemical recurrence or 18 months after radical prostatectomy. Patients were censored at last follow-up.

Endpoints were compared between the G-MINOR and matched MUSIC cohorts. Endpoint capture for G-MINOR used the existing MUSIC data registry, ensuring that there were no differences in outcome measurement. To further probe the association between clinical trial participation and clinical care, rates and time to secondary treatment were also compared by genomic risk strata (high, intermediate, low) under the same null hypothesis that management within a particular risk stratum should be similar between the G-MINOR and MUSIC cohorts. In this comparison, only G-MINOR patients for whom genomic classifier data were available to clinicians for decisions making (ie, genomic classifier arm) were included.

A sensitivity analysis performed additional sets of matching after stratifying patients by genomic classifier receipt. To illustrate, patients in the usual care arm of G-MINOR and patients from MUSIC not receiving genomic classifier were matched on clinicopathologic variables; separately, patients in the genomic classifier arm of G-MINOR and patients from MUSIC receiving genomic classifier were matched.

### Statistical analysis

Patient characteristics were first tabulated and compared between the G-MINOR and unmatched MUSIC cohorts using ꭓ^2^ or Kruskal-Wallis tests. Nearest-neighbor propensity score matching was then performed on clinically relevant variables (age, race, Charlson Comorbidity Index, pre–radical prostatectomy PSA level, Gleason Grade Group on radical prostatectomy, pathological T stage, and positive surgical margins) and compared before and after matching using standardized mean difference. Given the comparatively smaller period in the MUSIC “pre” cohort and the infrequent use of genomic classifier during this period, fewer patients were available, and matching was performed 1:1; otherwise, matching was performed 3:1.

The study hypothesis was then tested using comparison of Kaplan-Meier secondary treatment-free survival estimates between the G-MINOR and matched MUSIC cohorts using a log rank test with matching subclass included as a strata variable to account for the pairs in the matched sample. All tests used *P* < .05 to define statistical significance. Analyses and data visualization were performed in *R*, version 4.4.1, statistical software (R Foundation for Statistical Computing). MUSIC practices obtained exemption or approval from their institutional review boards before participation; the G-MINOR protocol and informed consent forms were institutional review board approved at all participating sites. This analysis was deemed exempt from review by the University of Michigan Institutional Review Board. All studies were performed according to the Declaration of Helsinki. The study followed the Strengthening the Reporting of Observational Studies in Epidemiology reporting guideline for cohort studies.

## Results

A total of 1842 and 554 non–G-MINOR–enrolled MUSIC patients met inclusion criteria at trial sites in the during-trial and pretrial periods and, along with 338 G-MINOR trial patients, made up the prematched study cohorts.

### Matched MUSIC cohort

Patients from the real-world MUSIC cohort had a lower proportion of pT3+ (67% vs 73%, *P* = .03) but a higher rate of positive surgical margins (66% vs 57%, *P* = .003) compared with G-MINOR patients but were otherwise similar (Table 1). After 3:1 nearest-neighbor matching, the standardized mean difference across all included variables was less than 0.1.

**Table 1.** Baseline characteristics of G-MINOR and the during-trial MUSIC cohort, before and after 3:1 nearest-neighbor propensity score matching.

	Before matching				After matching			
Characteristic	MUSIC	G-MINOR	***P*** ^a^	Standardized mean difference	MUSIC	G-MINOR	***P*** ^a^	Standardized mean difference
Patients, No.	1842	338			1014	338		
Age, mean (SD), y	63.5 (6.9)	63.9 (6.4)	.248	0.07	64 (6.5)	63.9 (6.4)	.84	0.012
Race, No. (%)			.13	0.202			.94	0.068
African American	236 (12.8)	24 (7.1)			65 (6.4)	24 (7.1)		
Asian	15 (0.8)	3 (0.9)			5 (0.5)	3 (0.9)		
White	1363 (74.0)	269 (79.6)			827 (81.6)	269 (79.6)		
American Indian or Alaska Native	6 (0.3)	1 (0.3)			4 (0.4)	1 (0.3)		
Pacific Islander	1 (0.1)	0 (0)			0 (0)	0 (0)		
Other	32 (1.7)	4 (1.2)			11 (1.1)	4 (1.2)		
Unknown	189 (10.3)	37 (10.9)			102 (10.1)	37 (10.9)		
Charlson Comorbidity Index, mean (SD)	0.34 (0.72)	0.39 (0.78)	.237	0.068	0.35 (0.77)	0.39 (0.78)	.452	0.047
Pre–radical prostatectomy PSA level, mean (SD), ng/mL	10.04 (26.24)	7.61 (4.73)	.089	0.129	7.93 (7.16)	7.61 (4.73)	.431	0.054
Gleason Grade Group, No. (%)			.209	0.15			.869	0.07
1	76 (4.1)	15 (4.4)			54 (5.3)	15 (4.4)		
2	860 (46.7)	171 (50.6)			530 (52.3)	171 (50.6)		
3	509 (27.6)	98 (29.0)			285 (28.1)	98 (29)		
4	152 (8.3)	18 (5.3)			44 (4.3)	18 (5.3)		
5	245 (13.3)	36 (10.7)			101 (10.0)	36 (10.7)		
Pathologic T-stage pT3+, No. (%)	1227 (66.6)	246 (72.8)	.03	0.135	758 (74.8)	246 (72.8)	.518	0.045
Positive surgical margins, No. (%)	1214 (65.9)	194 (57.4)	.003	0.176	601 (59.3)	194 (57.4)	.588	0.038
Extraprostatic extension, No. (%)	1161 (63)	234 (69.2)	.034	0.131	718 (70.8)	234 (69.2)	.63	0.034

Abbreviations: G-MINOR = Genomics in Michigan Impacting Observations of Radiation; MUSIC = Michigan Urological Surgery Improvement Collaborative; PSA = prostate-specific antigen.

a *P* values represent the results of ꭓ^2^ or Kruskal-Wallis test for categorical and ordinal variables, respectively.

All 338 patients in G-MINOR and 1014 matched patients in MUSIC were then compared for study endpoints. Over a median follow-up of 3.1 years, 69 G-MINOR patients received secondary treatment (31 adjuvant, 38 salvage) compared with 266 MUSIC patients (cohort median follow-up = 2.9 years: 183 adjuvant, 83 salvage).

**Figure 2.**
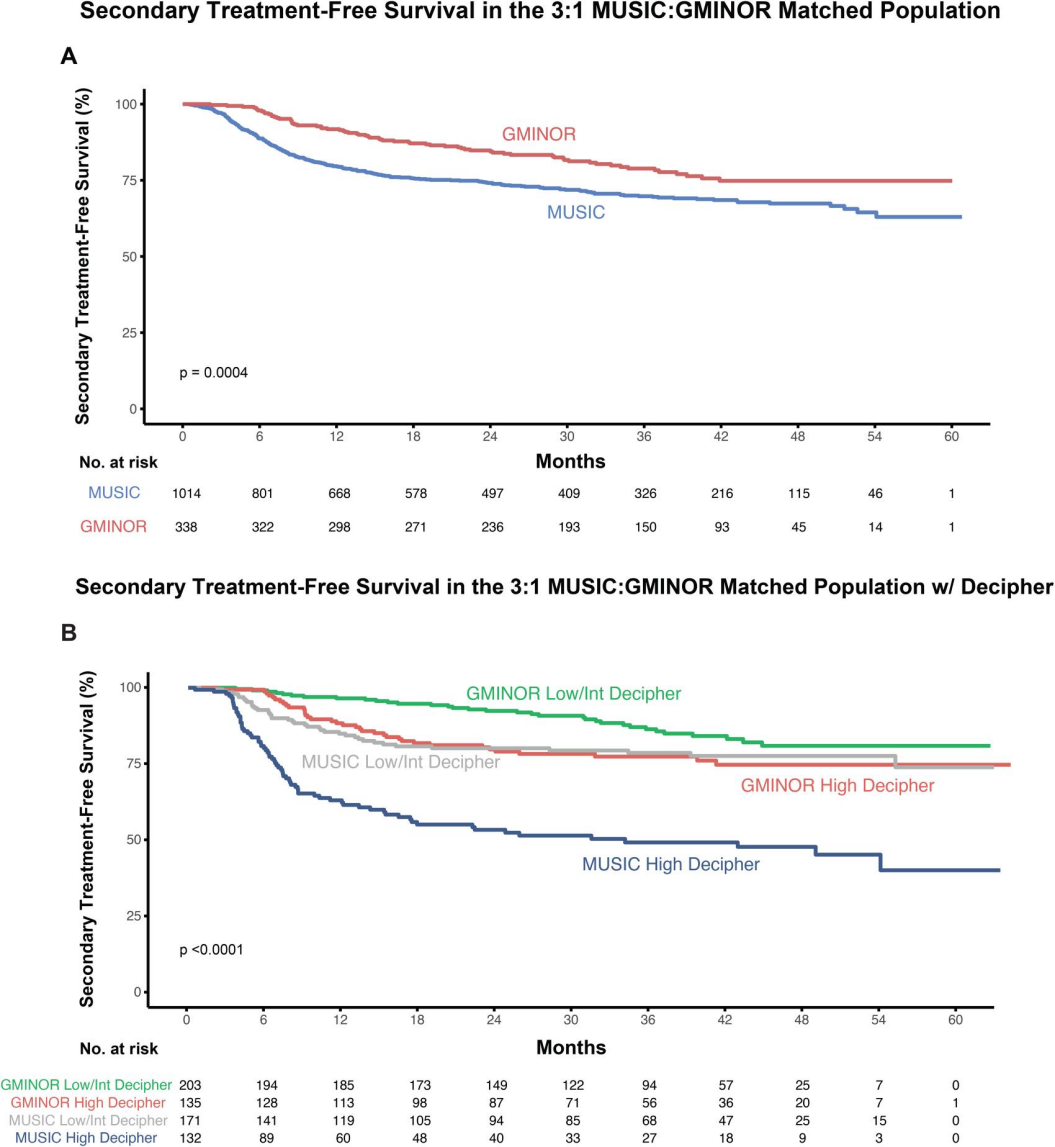
Kaplan-Meier estimates of secondary treatment-free survival. G-MINOR and during-trial MUSIC cohorts are compared in (**A**) and stratified by genomic classifier risk (**B**). *P* values are from log rank tests, with matching subclass included as a strata variable to account for pairs in the matched samples in (**A**) and log rank test in (**B**). G-MINOR = Genomics in Michigan Impacting Observations of Radiation; MUSIC = Michigan Urological Surgery Improvement Collaborative.

Kaplan-Meier estimates of time to secondary treatment demonstrated a statistically significant difference between the G-MINOR (2-year treatment-free survival = 84%) and MUSIC cohorts (2-year treatment-free survival = 74%; *P* < .001) (Figure 2, A).

Cohorts were stratified by genomic classifier risk group (high vs intermediate/low) (Figure 2, B). Again, time to secondary treatment was shorter among MUSIC patients (2-year treatment-free survival high risk = 44%; intermediate/low risk = 77%) compared with G-MINOR patients (2-year treatment-free survival high risk = 76%; intermediate/low risk = 91%, *P* < .001 across all groups) at a given genomic classifier risk group.

New matches were then performed after stratifying MUSIC and G-MINOR by receipt of genomic classifier testing (genomic classifier arm [G-MINOR] and genomic classifier receipt [MUSIC]; usual care arm and no genomic classifier receipt). Unmatched and matched cohort characteristics are shown in Tables S1 and S2; standardized mean differences among matched cohorts were less than 0.2 for all variables. Across both strata (genomic classifier, no genomic classifier), time to secondary treatment was shorter among MUSIC patients than among G-MINOR patients (2-year treatment-free survival genomic classifier: G-MINOR = 82%, MUSIC = 66%, *P* = .001; no genomic classifier: G-MINOR = 86%, MUSIC = 78%, *P* = .016) (Figure S1).

### Matched MUSIC pretrial cohort

Patients in the pretrial MUSIC cohort were younger (mean age = 63 vs 64 years, *P* = .033), more likely to be African American (14.3% vs 7.1%, *P* = .002 across race categories), and had a higher pre–radical prostatectomy PSA level (mean [SD] = 8.96 [8.82] vs 7.61 [4.73] ng/mL, *P* = .009) than did G-MINOR patients (Table S3). Characteristics of both groups after 1:1 nearest-neighbor matching are shown in Table 2; standardized mean differences across included variables were all below 0.2.

**Table 2.** Baseline characteristics of the G-MINOR and pretrial MUSIC cohort before and after 3:1 nearest-neighbor propensity score matching.

	Before matching				After matching			
	MUSIC	G-MINOR	***P*** ^a^	Standardized mean difference	MUSIC	G-MINOR	***P*** ^a^	Standardized mean difference
Patients, No.	554	338			338	338		
Age, mean (SD), y	62.9 (6.7)	63.9 (6.4)	.03	0.148	64 (6.2)	63.9 (6.4)	.865	0.013
Race, No. (%)			.002	0.322			.779	0.122
African American	79 (14.3)	24 (7.1)			28 (8.3)	24 (7.1)		
Asian	4 (0.7)	3 (0.9)			3 (0.9)	3 (0.9)		
White	429 (77.4)	269 (79.6)			275 (81.4)	269 (79.6)		
American Indian or Alaska Native	0 (0)	1 (0.3)			0 (0)	1 (0.3)		
Pacific Islander	3 (0.5)	0 (0)			0 (0)	0 (0)		
Other	8 (1.4)	4 (1.2)			3 (0.9)	4 (1.2)		
Unknown	31 (5.6)	37 (10.9)			29 (8.6)	37 (10.9)		
Charlson Comorbidity Index, mean (SD)	0.32 (0.71)	0.39 (0.78)	.205	0.087	0.36 (0.73)	0.39 (0.78)	.647	0.035
Pre–radical prostatectomy PSA level, mean (SD), ng/mL	8.96 (8.82)	7.61 (4.73)	.009	0.192	7.69 (4.98)	7.61 (4.73)	.831	0.016
Gleason Grade Group, No. (%)			.223	0.168			.956	0.062
1	37 (6.7)	15 (4.4)			14 (4.1)	15 (4.4)		
2	248 (44.8)	171 (50.6)			171 (50.6)	171 (50.6)		
3	159 (28.7)	98 (29.0)			103 (30.5)	98 (29.0)		
4	44 (7.9)	18 (5.3)			14 (4.1)	18 (5.3)		
5	66 (11.9)	36 (10.7)			36 (10.7)	36 (10.7)		
Pathologic T-stage pT3+, No. (%)	387 (69.9)	246 (72.8)	.391	0.065	243 (71.9)	246 (72.8)	.863	0.02
Positive surgical margins, No. (%)	330 (59.6)	194 (57.4)	.57	0.044	196 (58.0)	194 (57.4)	.938	0.012
Extraprostatic extension, No. (%)	365 (65.9)	234 (69.2)	.338	0.072	230 (68.0)	234 (69.2)	.804	0.026

Abbreviations: G-MINOR = Genomics in Michigan Impacting Observations of Radiation; MUSIC = Michigan Urological Surgery Improvement Collaborative; PSA = prostate-specific antigen.

a *P* values represent the results of ꭓ^2^ or Kruskal-Wallis test for categorical and ordinal variables, respectively.

**Figure 3.**
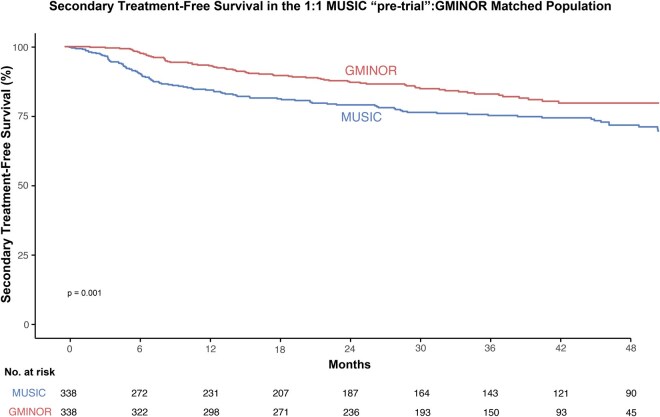
Kaplan-Meier estimates of secondary treatment-free survival, comparing overall G-MINOR and pretrial matched MUSIC cohorts. The *P* value is from the log rank test, with matching subclass included as a strata variable to account for pairs in the matched samples. G-MINOR = Genomics in Michigan Impacting Observations of Radiation; MUSIC = Michigan Urological Surgery Improvement Collaborative.

All 338 patients in G-MINOR and 338 matched patients in the pretrial MUSIC cohort were then compared for study endpoints. Over a median follow-up of 3.7 years, 104 MUSIC patients received secondary treatment (60 adjuvant, 44 salvage). Kaplan-Meier estimates of time to secondary treatment demonstrated a statistically significant difference between the G-MINOR (2-year treatment-free survival = 84%) and MUSIC cohorts (2-year treatment-free survival = 74%; *P* = .001) (Figure 3).

Finally, an additional match was performed after stratifying by receipt of genomic classifier testing. Given the low number of patients in the temporally condensed pretrial cohort and limited rates of genomic classifier testing before the trial, matching was performed only among patients who did not receive genomic classifier testing (usual care arm of G-MINOR or MUSIC cohort without genomic classifier testing). Unmatched and matched characteristics are shown in Table S3; standardized mean differences across all variables was less than 0.2. Time to secondary treatment was shorter among MUSIC patients than among G-MINOR patients (2-year treatment-free survival: G-MINOR = 86%, MUSIC = 73%; *P* = .012) (Figure S2).

## Discussion

In this study, we compared rates of post–radical prostatectomy secondary treatment in patients at high risk of disease recurrence in a prospective randomized controlled trial vs matched nonrandomized controlled trial cohorts. Importantly, we drew our patients with identical inclusion criteria from a statewide clinical registry in which the randomized controlled trial of interest, G-MINOR, was embedded, allowing for the identification of a real-world cohort that controlled for clinician factors and data infrastructure considerations. Further, we assembled 2 separate real-world cohorts and performed matching on clinicopathologic variables to minimize confounding. Our results support a hypothesis that, in the context of post–radical prostatectomy prostate cancer treatment, clinicians manage clinical trial patients differently from their real-world counterparts across multiple decision types (ie, naive and informed regarding genomic classifier risk), administering secondary treatment more selectively within a trial setting. Few studies have robustly compared intervention and outcomes between randomized controlled trial and nonrandomized controlled trial settings from the same patient and clinician populations; none, based on a review of the literature, involves a clinician decision-making endpoint the way G-MINOR did. Specifically, this clinician decision-making endpoint comparison has substantial implications for the conduct and generalizability of contemporary clinical trials. Pervasive differences between characteristics and care received across contexts argues against the notion that treatment effect estimates generated from randomized controlled trial evidence not re-created in real-world cohorts are invalid. Populations are intrinsically different in a way that makes real-world “validation” through re-creation of trial conditions irrelevant.

Our results provide context to the notion of whether randomized controlled trial participation improves clinical outcomes regardless of patient allocation or intervention efficacy.^18^^,^^19^ This notion had previously been supported by the National Comprehensive Cancer Network and others, stating that trial enrollment is the best care of a patient with cancer, but refuted by a recent meta-analysis by Iskander et al.^20^ that used strict study inclusion criteria. Their work focused on the impact of pharmaceutical trial participation on overall survival, a relatively distal outcome subject to influences beyond a trial’s infrastructure. They identified physician behavior as just 1 possible mechanism by which trial participation may influence survival, along with effects of patient monitoring, patient expectations, and prognostic confounders. Our study, by assessing the generalizability of a trial focused on clinician decision making, was uniquely positioned to explore the influence of trial participation specifically on clinician behavior (ie, administration of secondary treatment). We found a substantial difference in rates of secondary treatment between the trial and real-world populations distinct from the aforementioned meta-analysis, which did not identify a substantial influence of trial participation on overall survival. Whether randomized controlled trial participation affects survival may be highly context dependent. Here, we provide 1 rigorous example of how trial participation directly influences patient management, illustrating a measurable influence of the Hawthorne Effect. As follow-up data continue to mature for both G-MINOR and MUSIC, the impact of trial participation on long-term oncological outcomes will be further elucidated.

Although the Hawthorne Effect may fit our findings, an alternative hypothesis is that mandatory use of the CAPRA-S risk score accounts for the observed discrepancy in secondary treatment because this is not a required, standard-of-care practice in the real-world setting. Thus, the usual care/control arm of G-MINOR consisted of risk stratification using CAPRA-S, a practice that may not reflect typical care in the community. There are no prospective studies on the impact of the CAPRA-S score alone on management decisions following prostatectomy. It is plausible that simply presenting clinicians with an objective, quantitative risk estimate may alter their decision-making process. Although clinical vignette research from other surgical specialties suggests that risk scores do recalibrate clinician risk estimates, actual decisions are less likely to change, supporting the notion that the observed, discrepant management is a product of changed behavior under observation.^21^

Finally, our results draw attention to the interpretation of the G-MINOR trial’s primary analysis. The care of high-risk post–radical prostatectomy patients with respect to secondary treatment is an actively changing landscape. Tissue-based gene expression classifiers are positioned to inform management, but their actual impact on clinical practice was previously unknown. Primary analysis of the G-MINOR trial revealed similar rates of adjuvant treatment between arms but a higher allocation of this treatment to high-risk patients following genomic classifier testing.^15^ Although our results show different secondary treatment patterns within and outside the trial, the relationship of differential management based on risk grouping by genomic classifier is preserved across cohorts, supporting the notion that genomic classifier testing may reallocate treatment to higher-risk patients.

This study is not without limitations. As mentioned earlier, we are unable to discriminate between the Hawthorne Effect and the impact of risk stratification by CAPRA-S score on treatment. Furthermore, although site matching and propensity score matching control for a substantial amount of bias, it is possible that the observed effect is due to unmeasured, uncontrolled confounding given the post hoc nature of the analysis. Finally, our observation of divergent management may be specific to the clinical scenario under study and does not necessarily reflect the notion that randomized controlled trial care differs universally from the real world.

Nonetheless, we show substantially different secondary treatment patterns following radical prostatectomy between typical MUSIC patients and patients enrolled in the G-MINOR randomized controlled trial. These results shed further light on the challenges associated with trial design, generalizability, and translation into clinical practice. Future work will elucidate mechanisms for increasing the real-world fidelity of randomized controlled trial conduct and results.

## Supplementary Material

pkag040_Supplementary_Data

## Data Availability

The data sets generated during the current study are not publicly available because they are institutional. They are available, however, from the corresponding author on reasonable request.
