# Supplementary material for: Understanding randomized controlled trial generalizability through an embedded molecular diagnostics trial
Source: JNCI Cancer Spectr. 2026 Apr 24;10(3):pkag040. doi: 10.1093/jncics/pkag040 (PMC13226051; doi:10.1093/jncics/pkag040)
Supplement: pkag040_Supplementary_Data [file pkag040_supplementary_data.zip › Supplementary Tables.docx]

|  | **Before Match** | | | |  | **After Match** | | | |
| --- | --- | --- | --- | --- | --- | --- | --- | --- | --- |
| Characteristic | MUSIC | GMINOR | p-value | SMD |  | MUSIC | GMINOR | p-value | SMD |
| **Number of patients** | 229 | 175 |  |  |  | 175 | 175 |  |  |
| **Age (mean (SD))** | 62.97 (6.82) | 63.79 (6.52) | 0.223 | 0.123 |  | 63.78 (6.47) | 63.79 (6.52) | 0.98 | 0.003 |
| **Race (%)** |  |  | 0.131 | 0.296 |  |  |  | 0.494 | 0.198 |
| African American | 32 (14.0) | 15 ( 8.6) |  |  |  | 16 ( 9.1) | 15 ( 8.6) |  |  |
| Asian | 3 ( 1.3) | 1 ( 0.6) |  |  |  | 2 ( 1.1) | 1 ( 0.6) |  |  |
| Caucasian | 178 (77.7) | 136 (77.7) |  |  |  | 144 (82.3) | 136 (77.7) |  |  |
| Native American | 1 ( 0.4) | 0 ( 0.0) |  |  |  | 0 ( 0.0) | 0 ( 0.0) |  |  |
| Pacific Islander | 0 ( 0.0) | 0 ( 0.0) |  |  |  | 0 ( 0.0) | 0 ( 0.0) |  |  |
| Other | 2 ( 0.9) | 4 ( 2.3) |  |  |  | 2 ( 1.1) | 4 ( 2.3) |  |  |
| Unknown / Refused | 13 ( 5.7) | 19 (10.9) |  |  |  | 11 ( 6.3) | 19 (10.9) |  |  |
| **CCI (mean (SD))** | 0.35 (0.66) | 0.43 (0.76) | 0.29 | 0.105 |  | 0.39 (0.68) | 0.43 (0.76) | 0.606 | 0.055 |
| **PSA Before RP (mean (SD))** | 7.71 (6.81) | 8.04 (5.20) | 0.587 | 0.056 |  | 7.45 (4.65) | 8.04 (5.20) | 0.264 | 0.12 |
| **Grade Group (%)** |  |  | 0.602 | 0.17 |  |  |  | 0.982 | 0.068 |
| 1 | 10 ( 4.4) | 9 ( 5.1) |  |  |  | 7 ( 4.0) | 9 ( 5.1) |  |  |
| 2 | 113 (49.3) | 90 (51.4) |  |  |  | 93 (53.1) | 90 (51.4) |  |  |
| 3 | 65 (28.4) | 51 (29.1) |  |  |  | 52 (29.7) | 51 (29.1) |  |  |
| 4 | 20 ( 8.7) | 8 ( 4.6) |  |  |  | 7 ( 4.0) | 8 ( 4.6) |  |  |
| 5 | 21 ( 9.2) | 17 ( 9.7) |  |  |  | 16 ( 9.1) | 17 ( 9.7) |  |  |
| **Pathologic T Stage pT3+ (%)** | 168 (73.4) | 125 (71.4) | 0.75 | 0.043 |  | 128 (73.1) | 125 (71.4) | 0.811 | 0.038 |
| **Positive Margins (%)** | 146 (63.8) | 100 (57.1) | 0.212 | 0.136 |  | 106 (60.6) | 100 (57.1) | 0.587 | 0.07 |
| **EPE (%)** | 157 (68.6) | 122 (69.7) | 0.888 | 0.025 |  | 125 (71.4) | 122 (69.7) | 0.815 | 0.038 |

**Supplementary Table 1.** Baseline characteristics of patients in G-MINOR and “during trial” MUSIC cohorts who underwent GC testing (GC arm in trial, receipt of GC in MUSIC), before and after 1:1 nearest neighbor propensity score matching. P-values represent results of chi-squared or Kruskal-Wallis tests for categorical and ordinal variables, respectively. SMD: standardized mean difference.

|  | **Before Match** | | | |  | **After Match** | | | |
| --- | --- | --- | --- | --- | --- | --- | --- | --- | --- |
| Characteristics | MUSIC | GMINOR | p-value | SMD |  | MUSIC | GMINOR | p-value | SMD |
| **Number of patients** | 1613 | 163 |  |  |  | 489 | 163 |  |  |
| **Age (mean (SD))** | 63.52 (6.92) | 64.06 (6.28) | 0.344 | 0.081 |  | 64.13 (6.34) | 64.06 (6.28) | 0.898 | 0.012 |
| **Race (%)** |  |  | 0.072 | 0.332 |  |  |  | 0.791 | 0.105 |
| African American | 204 (12.6) | 9 ( 5.5) |  |  |  | 27 ( 5.5) | 9 ( 5.5) |  |  |
| Asian | 12 ( 0.7) | 2 ( 1.2) |  |  |  | 2 ( 0.4) | 2 ( 1.2) |  |  |
| Caucasian | 1185 (73.5) | 133 (81.6) |  |  |  | 410 (83.8) | 133 (81.6) |  |  |
| Native American | 5 ( 0.3) | 1 ( 0.6) |  |  |  | 2 ( 0.4) | 1 ( 0.6) |  |  |
| Pacific Islander | 1 ( 0.1) | 0 ( 0.0) |  |  |  | 0 ( 0.0) | 0 ( 0.0) |  |  |
| Other | 30 ( 1.9) | 0 ( 0.0) |  |  |  | 0 ( 0.0) | 0 ( 0.0) |  |  |
| Unknown / Refused | 176 (10.9) | 18 (11.0) |  |  |  | 48 ( 9.8) | 18 (11.0) |  |  |
| **CCI (mean (SD))** | 0.33 (0.73) | 0.34 (0.80) | 0.876 | 0.012 |  | 0.29 (0.65) | 0.34 (0.80) | 0.415 | 0.07 |
| **PSA Before RP (mean (SD))** | 10.37 (27.91) | 7.14 (4.12) | 0.14 | 0.162 |  | 8.93 (37.66) | 7.14 (4.12) | 0.545 | 0.067 |
| **Grade Group (%)** |  |  | 0.767 | 0.115 |  |  |  | 0.98 | 0.059 |
| 1 | 66 ( 4.1) | 6 ( 3.7) |  |  |  | 18 ( 3.7) | 6 ( 3.7) |  |  |
| 2 | 747 (46.3) | 81 (49.7) |  |  |  | 250 (51.1) | 81 (49.7) |  |  |
| 3 | 444 (27.5) | 47 (28.8) |  |  |  | 132 (27.0) | 47 (28.8) |  |  |
| 4 | 132 ( 8.2) | 10 ( 6.1) |  |  |  | 35 ( 7.2) | 10 ( 6.1) |  |  |
| 5 | 224 (13.9) | 19 (11.7) |  |  |  | 54 (11.0) | 19 (11.7) |  |  |
| **Pathologic T Stage pT3+ (%)** | 1059 (65.7) | 121 (74.2) | 0.034 | 0.188 |  | 379 (77.5) | 121 (74.2) | 0.454 | 0.077 |
| **Positive Margins (%)** | 1068 (66.2) | 94 (57.7) | 0.036 | 0.177 |  | 285 (58.3) | 94 (57.7) | 0.963 | 0.012 |
| **EPE (%)** | 1004 (62.2) | 112 (68.7) | 0.123 | 0.136 |  | 356 (72.8) | 112 (68.7) | 0.366 | 0.09 |

**Supplementary Table 2.** Baseline characteristics of patients in G-MINOR and “during trial” MUSIC cohorts who did not undergo GC testing (usual care arm in trial, no receipt of GC amongst MUSIC patients), before and after 3:1 nearest neighbor propensity score matching. P-values represent results of chi-squared or Kruskal-Wallis tests for categorical and ordinal variables, respectively. SMD: standardized mean difference.

|  | **Before Match** | | | |  | **After Match** | | | |
| --- | --- | --- | --- | --- | --- | --- | --- | --- | --- |
| Characteristics | MUSIC | GMINOR | p-value | SMD |  | MUSIC | GMINOR | p-value | SMD |
| **Number of patients** | 527 | 163 |  |  |  | 163 | 163 |  |  |
| **Age (mean (SD))** | 62.92 (6.70) | 64.06 (6.28) | 0.055 | 0.175 |  | 63.71 (6.32) | 64.06 (6.28) | 0.617 | 0.056 |
| **Race (%)** |  |  | 0.002 | 0.436 |  |  |  | 0.853 | 0.129 |
| African American | 79 (15.0) | 9 ( 5.5) |  |  |  | 9 ( 5.5) | 9 ( 5.5) |  |  |
| Asian | 4 ( 0.8) | 2 ( 1.2) |  |  |  | 1 ( 0.6) | 2 ( 1.2) |  |  |
| Caucasian | 403 (76.5) | 133 (81.6) |  |  |  | 135 (82.8) | 133 (81.6) |  |  |
| Native American | 0 ( 0.0) | 1 ( 0.6) |  |  |  | 0 ( 0.0) | 1 ( 0.6) |  |  |
| Pacific Islander | 3 ( 0.6) | 0 ( 0.0) |  |  |  | 0 ( 0.0) | 0 ( 0.0) |  |  |
| Other | 8 ( 1.5) | 0 ( 0.0) |  |  |  | 0 ( 0.0) | 0 ( 0.0) |  |  |
| Unknown / Refused | 30 ( 5.7) | 18 (11.0) |  |  |  | 18 (11.0) | 18 (11.0) |  |  |
| **CCI (mean (SD))** | 0.32 (0.70) | 0.34 (0.80) | 0.704 | 0.033 |  | 0.29 (0.65) | 0.34 (0.80) | 0.495 | 0.076 |
| **PSA Before RP (mean (SD))** | 9.04 (9.00) | 7.14 (4.12) | 0.009 | 0.272 |  | 7.42 (3.92) | 7.14 (4.12) | 0.532 | 0.069 |
| **Grade Group (%)** |  |  | 0.508 | 0.172 |  |  |  | 0.984 | 0.068 |
| 1 | 37 ( 7.0) | 6 ( 3.7) |  |  |  | 6 ( 3.7) | 6 ( 3.7) |  |  |
| 2 | 236 (44.8) | 81 (49.7) |  |  |  | 83 (50.9) | 81 (49.7) |  |  |
| 3 | 149 (28.3) | 47 (28.8) |  |  |  | 49 (30.1) | 47 (28.8) |  |  |
| 4 | 40 ( 7.6) | 10 ( 6.1) |  |  |  | 9 ( 5.5) | 10 ( 6.1) |  |  |
| 5 | 65 (12.3) | 19 (11.7) |  |  |  | 16 ( 9.8) | 19 (11.7) |  |  |
| **Pathologic T Stage pT3+ (%)** | 368 (69.8) | 121 (74.2) | 0.326 | 0.098 |  | 120 (73.6) | 121 (74.2) | 1 | 0.014 |
| **Positive Margins (%)** | 308 (58.4) | 94 (57.7) | 0.933 | 0.016 |  | 94 (57.7) | 94 (57.7) | 1 | <0.001 |
| **EPE (%)** | 346 (65.7) | 112 (68.7) | 0.531 | 0.065 |  | 116 (71.2) | 112 (68.7) | 0.717 | 0.054 |

**Supplementary Table 3.** Baseline characteristics of patients in G-MINOR and “pre-trial” MUSIC cohorts who did not undergo GC testing (usual care arm in trial, no receipt of GC amongst MUSIC patients), before and after 1:1 nearest neighbor propensity score matching. P-values represent results of chi-squared or Kruskal-Wallis tests for categorical and ordinal variables, respectively. SMD: standardized mean difference.
