# Supplementary material for: Understanding randomized controlled trial generalizability through an embedded molecular diagnostics trial
Source: JNCI Cancer Spectr. 2026 Apr 24;10(3):pkag040. doi: 10.1093/jncics/pkag040 (PMC13226051; doi:10.1093/jncics/pkag040)

Supp Fig. 1a

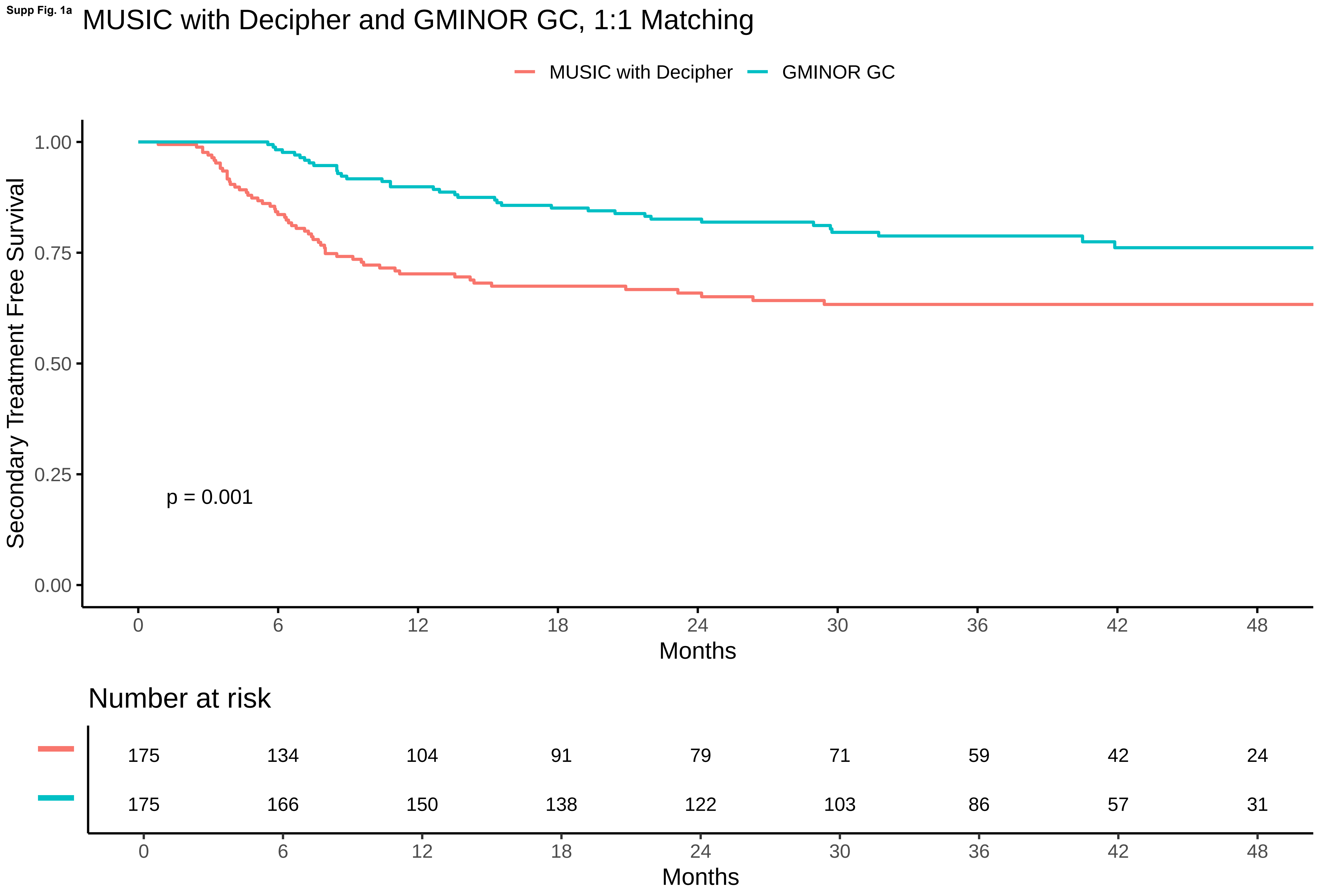
MUSIC with Decipher and GMINOR GC, 1:1 Matching
MUSIC with Decipher
GMINOR GC
Secondary Treatment Free Survival
1.00
0.75
0.50
0.25
0.00
p = 0.001
0
6
12
18
24
30
36
42
48
Months
Number at risk
175 134 104 91 79 71 59 42 24
175 166 150 138 122 103 86 57 31
Months

Supp Fig. 1b

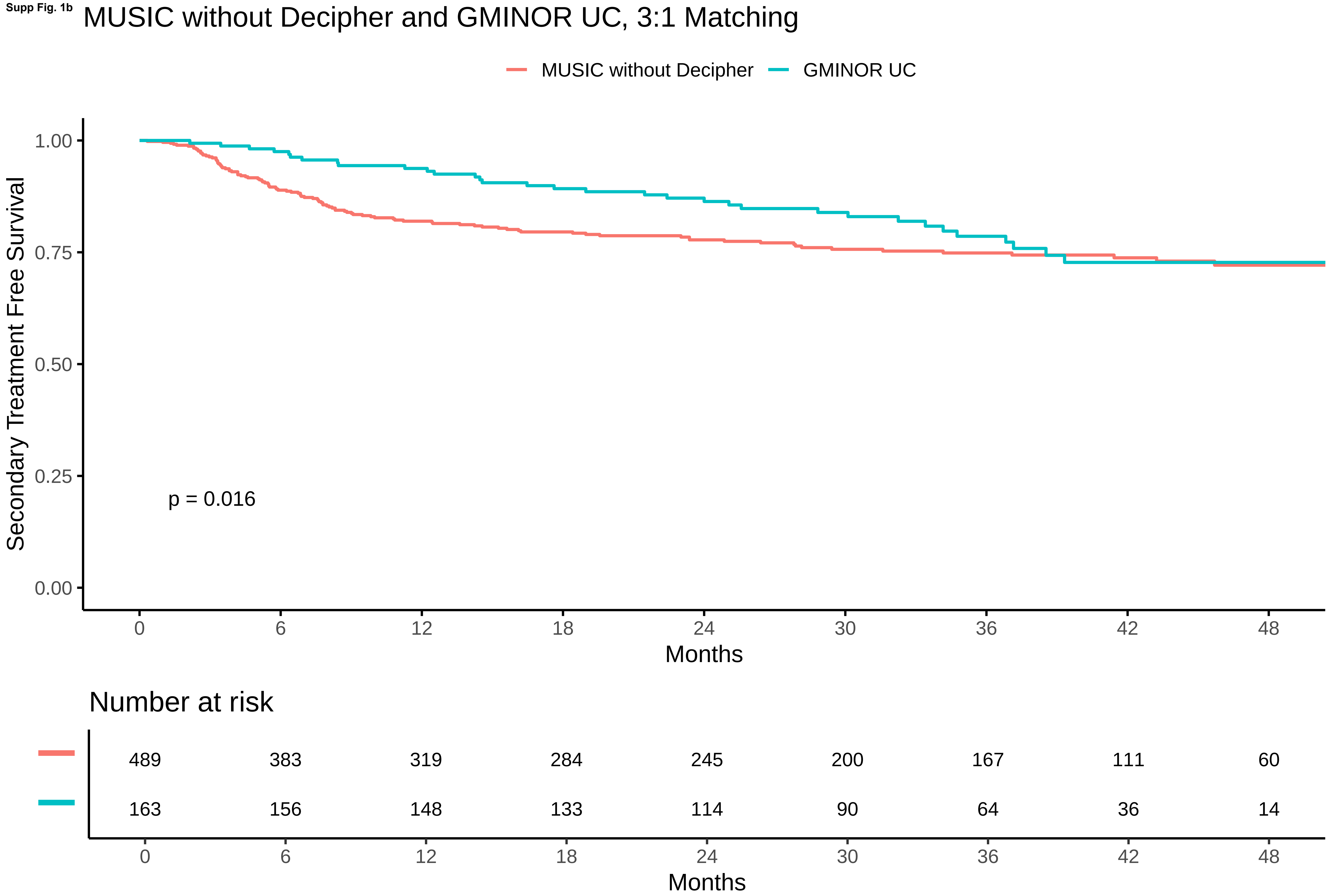

MUSIC without Decipher and GMINOR UC, 3:1 Matching
MUSIC without Decipher
GMINOR UC
Secondary Treatment Free Survival
1.00
0.75
0.50
0.25
0.00
p = 0.016
0
6
12
18
24
30
36
42
48
Months
Number at risk
489
383
319
284
245
200
167
111
60
163
156
148
133
114
90
64
36
14
0
6
12
18
24
30
36
42
48
Months

Supp Fig. 2

MUSIC (Before 3/2016) without Decipher and GMINOR UC, 1:1 Matching

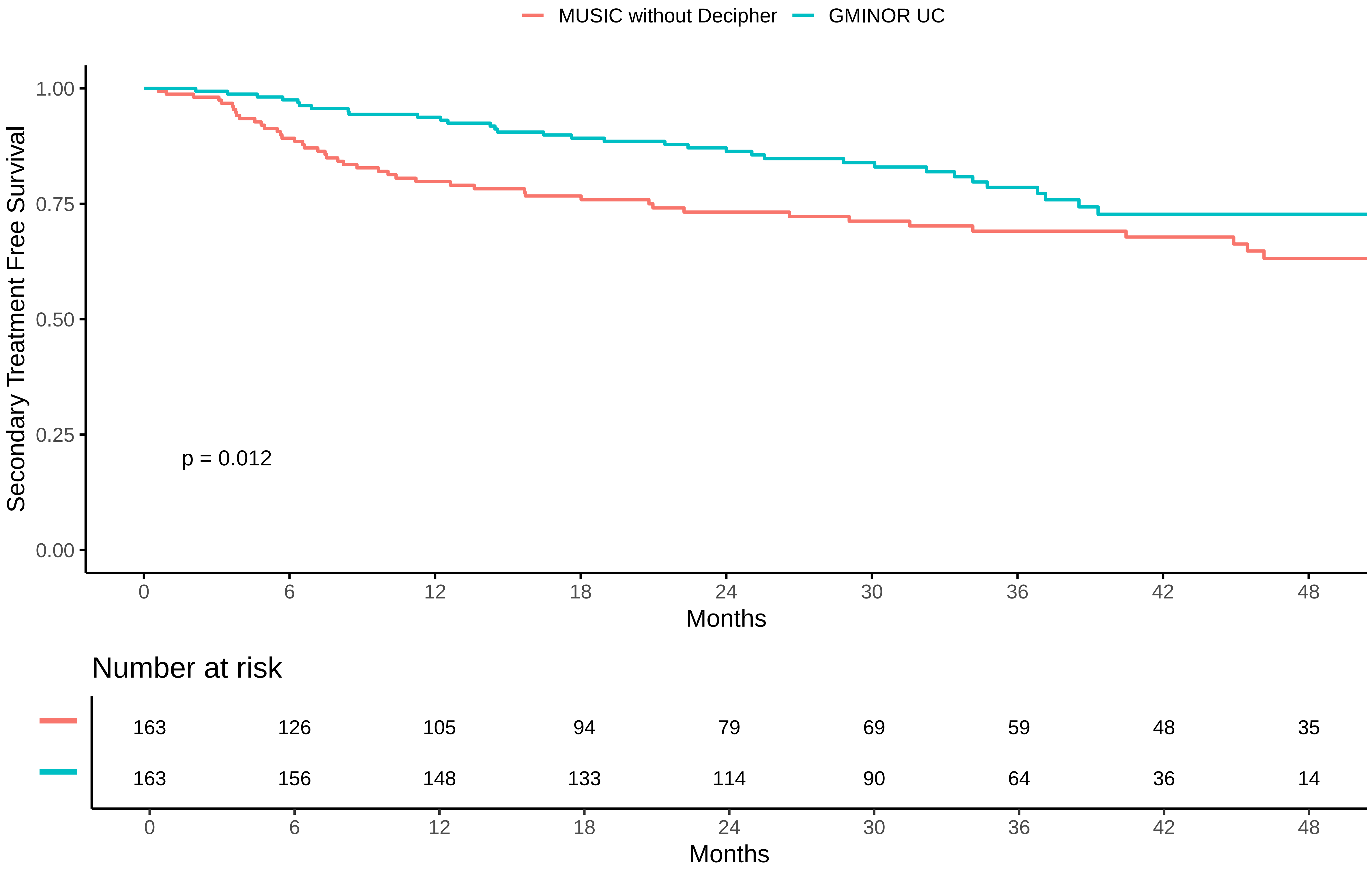

Supplement: pkag040_Supplementary_Data [file pkag040_supplementary_data.zip › Supplementary Figs.pdf]
